# Pyoderma gangrenosum: a 22-year follow-up of patients in a tertiary reference hospital in Brazil^☆^

**DOI:** 10.1016/j.abd.2024.07.014

**Published:** 2025-04-17

**Authors:** Livia Maria Oliveira Salviano, Denise Miyamoto, Claudia Giuli Santi, Tatiana Mina Yendo, Maria Cecilia Rivitti-Machado

**Affiliations:** Departament of Dermatology, Clinical Hospital, Faculty of Medicine, Universidade de São Paulo, São Paulo, SP, Brazil

**Keywords:** Pyoderma gangrenosum, Sweet syndrome, Skin ulcer

## Abstract

**Background:**

Pyoderma gangrenosum (PG) is a rare dermatosis often associated with systemic diseases. There are autoinflammatory mechanisms with neutrophilic infiltrate and necrosis. Due to the high morbidity, variable response to therapy and lack of treatment standardization, PG constitutes a highly burdensome condition.

**Objectives:**

To evaluate the epidemiological, clinical and laboratory features and response to therapy of patients with PG followed at HCFMUSP.

**Methods:**

This retrospective and descriptive study included patients with confirmed PG under follow-up at HCFMUSP from January 2000 to August 2021. Data were retrieved from medical records.

**Results:**

Fifty patients were included. The mean time from the onset of symptoms to diagnosis was 26.5 months. Lesions predominated on the lower extremity in 72% (n = 36/50), and the ulcerative type was the most common (n = 43/50; 86%). Local pain was mentioned in 39/50 (78%) and 12/50 (24%) presented pathergy. The most frequently associated diseases were inflammatory bowel disease (n = 10/20; 20%) and hidradenitis suppurativa (n = 10/20; 20%). High-dose systemic corticosteroid was mostly the first therapy (88%), either alone (n = 7/50; 14%) or in association with classic immunosuppressants or immunobiologicals (n = 37; 74%). Most patients (n = 32/50; 64%) had at least one hospitalization. Disease control was achieved in 44/50 (88%), with recurrences in 48% (n = 24/50) and total healing without medication in 24% (n = 12). Sixteen patients (32%) were treated with at least 1 immunobiological agent in addition to classic drugs.

**Study limitations:**

Retrospective, descriptive design and number of patients.

**Conclusions:**

There was delay in diagnosis, association with systemic and cutaneous conditions, and the need for prolonged immunomodulatory or immunosuppressive therapy (classic agents and also biologic agents) to control PG.

## Introduction

Pyoderma gangrenosum (PG) is an uncommon dermatosis, characterized by the accumulation of neutrophils in the dermis, hypodermis, and, rarely, in internal organs.^1^ PG affects 3‒10 people per million per year,^1^ especially adult women, but can occur at any age.^2^ PG lesions are considered an autoinflammatory manifestation resulting from dysregulation of innate immunity and overproduction of inflammatory mediators (such as interleukins 1 and 17, tumor necrosis factor-alpha) that promote tissue neutrophilic infiltration.^1^, ^3^

Clinical presentation of PG is polymorphous and includes the ulcerative (or classic), pustular, vegetative, and bullous (or atypical) variants. The ulcerative form is the most common, evolving from papules or pustules into painful ulcers with well-defined violaceous border and undermined edge. It mainly affects the lower limbs and is often associated with inflammatory bowel disease (IBD), inflammatory arthropathies, IgA gammopathy and neoplasms. The pustular variety, also associated with IBD, is characterized by pustules on the trunk and lower limbs that regress without scarring or may progress into classic PG. The vegetative form presents as a slow-growing single violaceous plaque or abscess, usually on the trunk, which heals with a cribriform pattern. Bullous PG is characterized by rapidly progressing blisters usually on the face and upper limbs, that progress to necrosis. It is frequently associated with hematologic diseases such as myeloid leukemia, lymphoma, monoclonal gammopathy, and myelodysplastic syndrome. Chronic PG that affects the oral and labial mucosa is called pyostomatitis vegetans. PG may also develop in other organs including lungs, kidneys, bones and eyes.^4^

PG may also be associated with HIV and hepatitis C infections, systemic lupus erythematosus, diabetes mellitus and psoriasis.^5^, ^6^ It may also be induced by drugs such as cocaine, propylthiouracil and antipsychotics. It may also occur in the context of autoinflammatory syndromes, such as PAPA (pyogenic arthritis, PG and acne), SAPHO (synovitis, acne, pustulosis, hyperostosis and osteitis) and PASH (PG, acne and hidradenitis suppurativa). Traumas, including surgical procedures, may worsen or trigger lesions, a phenomenon called pathergy. Postoperative pyoderma gangrenosum has been documented after orthopedic, cardiothoracic, general, plastic, and gynecological surgery. The challenge in diagnosis arises from its lack of recognition, its tendency to mimic wound infections and exacerbation after debridement (a standard practice for treating wound infections) due to pathergy.^6^, ^7^, ^8^, ^9^, ^10^, ^11^, ^12^, ^13^, ^14^, ^15^, ^16^, ^17^, ^18^, ^19^, ^20^, ^21^, ^22^, ^23^, ^24^, ^25^, ^26^

Once PG is suspected, a comprehensive evaluation is recommended including cutaneous biopsies for histopathological examination and cultures to rule out infectious, neoplastic, and autoimmune etiologies. The histopathology of PG is not specific showing mixed inflammatory infiltrate. Systemic workup includes the investigation of frequently associated diseases.^8^, ^9^, ^10^, ^11^, ^12^

Therapy varies according to PG severity, extension, associated diseases and patient tolerance, with the aim of reducing inflammatory activity, promoting wound healing, pain control and management of associated conditions. In addition, traumas such as surgical debridement and grafting should be carefully indicated, as they may worsen or trigger new lesions.^1^, ^10^ Systemic corticosteroids and cyclosporine are considered first-line therapy. Other immunosuppressants and immunobiological agents are often required due to the refractoriness of PG lesions.^8^, ^12^, ^27^

PG portends a high impact on patient's quality of life either because of the painful and disfiguring lesions or due to the associated comorbidities.^4^ The delay in diagnosis due to the rarity of the disease and the inexperience of health professionals, the socioeconomic burden related to the time spent with medical care, adverse drug effects and hospital admissions further contribute to increased PG morbidity.^1^, ^3^, ^9^

The objective of this study is to outline the clinical, epidemiological, laboratory and therapeutic profile of patients with PG under follow-up at Hospital das Clínicas, Faculdade de Medicina, Universidade de São Paulo (University of São Paulo Medical School Hospital) from January 2000 to August 2021.

## Methods

After ethics committee approval (CAAE: 57067622.0.0000.0068), this retrospective and descriptive study included all patients with PG under follow-up at the Dermatology Department of HCFMUSP from January 2000 to August 2021. The diagnosis of PG was based on (1) clinical features, (2) compatible histopathological findings and (3) exclusion of other differential diagnoses.

Clinical records of patients were analyzed to obtain the following data: sociodemographic variables, previous diagnostic hypotheses, onset of symptoms, number and location of lesions, clinical subtypes, associated symptoms, comorbidities, laboratory and histopathological exams, medications, hospitalizations, time to achieve disease control and recurrences.

Categorical variables were expressed as frequencies and percentages. Means, standard deviation, median and minimum and maximum values were calculated for quantitative variables. The association between categorical variables was assessed using Pearson's Chi-Square test. The significance level adopted was 5%. Analyzes were performed using SPSS for Windows v.25 statistical software.

## Results

### Sociodemographic data

From January 2000 to August 2021, 50 patients were diagnosed with PG. There was a female predominance (n = 29/50; 58%). The mean age at the onset of symptoms was 33.7 years (3 to 65 years); 87.8% declared themselves white. Other sociodemographic characteristics are presented in Table 1.

**Table 1:** Gender, age at onset and skincolor of patients.

Aspects	n = 50		n (%)	n_missing_
*Gender*	Female		29 (58.0)	
	Male		21 (42.0)	
*Age at onset (years)*	Means (SD)	33.7 (16.0)		
	Median (min-max)	30 (3-65)		
*skin color (self-declared)*	White		43 (87.8)	1
	Black		4 (8.2)	
	Brown		2 (4.1)	

### Clinical features and comorbidities

PG was the single diagnostic hypothesis in 24 out of 50 (48%) cases. Mean age at diagnosis was 36.2 years (5 to 65-years), with a mean duration of symptoms of 26.5 months (0.25 to 480-months).

**Figure 1:**
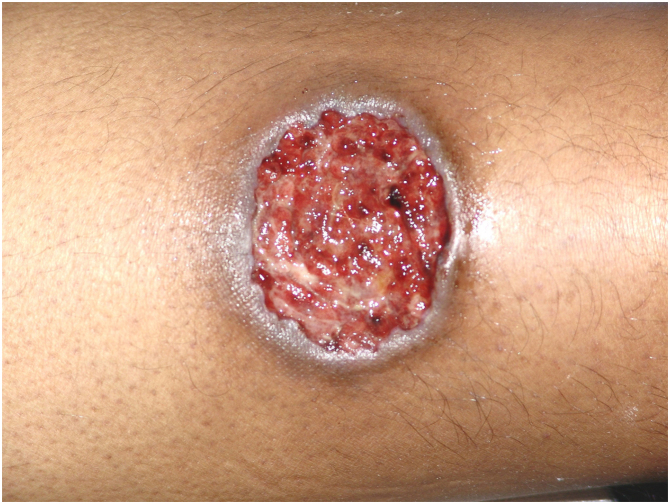
Clinical subtype of pyoderma gangrenosum – ulcerative.

**Figure 2:**
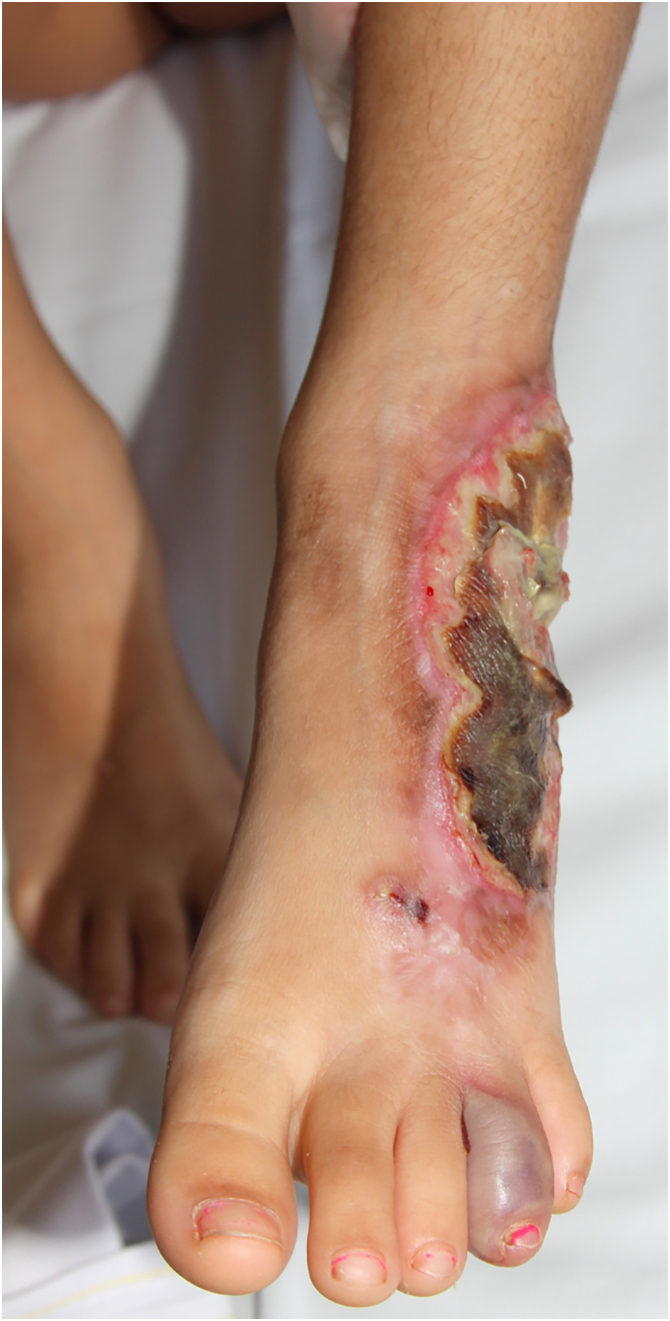
Clinical subtype of pyoderma gangrenosum – bullous.

**Figure 3:**
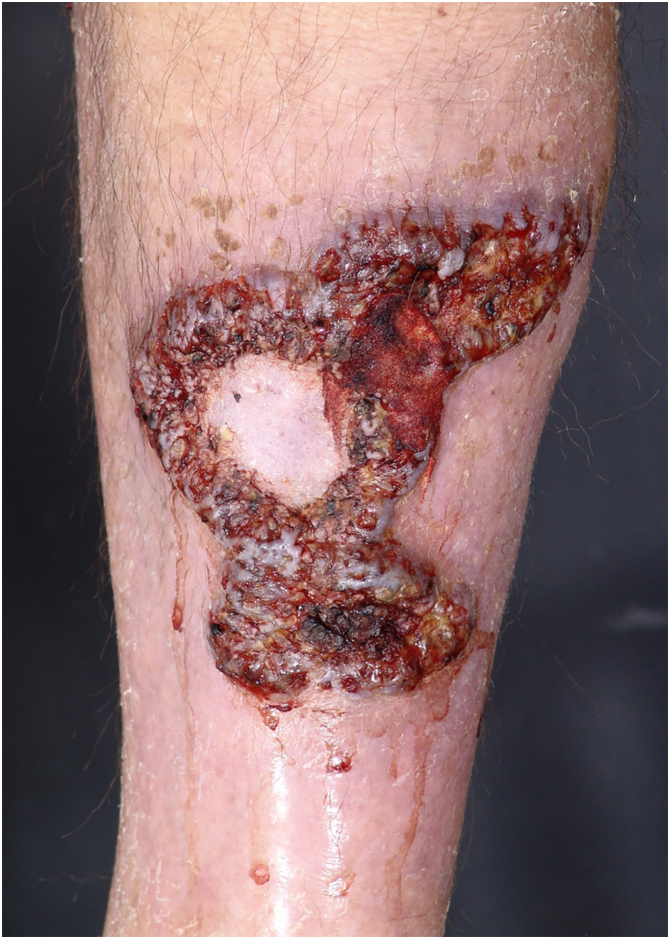
Clinical subtype of pyoderma gangrenosum – vegetative.

Multiple lesions (> 2) occurred in 19 out of 50 (38%) patients. The most frequent region was the lower extremity (n = 36/50; 72%), followed by the upper extremity (n = 15/50; 30%), trunk (n = 13/50; 26%) and head and neck (n = 12/50; 24%). The ulcerative subtype (Fig. 1) was the most common (n = 43/50; 86%) (Table 2). Two patients had oral involvement and one had systemic involvement (lungs). Twenty patients (40%) had lesions in more than one location, and the association of lower and upper limbs was the most frequent one (n = 7/20; 35%). When multiple lesions were presented, the ulcerative subtype was the most found (n = 17/20; 85%). Bullous and vegetative subtypes are illustrated in Figure 2, Figure 3.

**Table 2:** Number, location and subtype of lesions.

Aspects	n = 50	n (%)
Number of lesions at onset	1 lesion	18 (36.0)
	2 lesions	13 (26.0)
	>2 lesions	19 (38.0)
**Location**		
Lower limbs		36 (72.0)
Upper limbs		15 (30.0)
Head and Neck		12 (24.0)
Trunk		13 (26.0)
Oral		2 (4.0)
Other location	Breast; Genital	2 (4.0)
**Clinical subtype**		
Ulcerative		43 (86.0)
Pustular		1 (2.0)
Bullous		1 (2.0)
Vegetative		7 (14.0)
Pyostomatitis vegetans		2 (4.0)
Systemic Involvement	Pulmonar	1 (2.0)

Local pain, usually of extreme intensity was present in 39 (58% of the cases). Systemic symptoms were present in 40 (80%) cases: fever in 12 (24%), joint pain in 11 (22%) and weight loss in 7 (14.9%).

The associated comorbidities are detailed in Table 3.

**Table 3:** Associated conditions.

Aspects	n = 50 (%)
Associated conditions	40 (80.0)
***Inflammatory bowel disease***	10 (20.0)
Crohn's disease	3 (6.0)
Ulcerative colitis	7 (14.0)
***Solid organ neoplasm***	2 (4.0)
Prostate	1
Liver	1
***Hematological disease***	5 (10.0)
Monoclonal gammopathy	1
Policlonal gammopathy	1
Myelofibrosis	1
Myelodysplastic syndrome	2
***Arthritis***	6 (12.0)
Lupus arthritis	1
Psoriatic arthritis	1
Rheumatoid arthritis	3
Pyogenic arthritis	1
***Hidradenitis suppurativa***	10 (20.0)
PASH	2
PAPASH	2
**PAPA**	1
**Undefined autoinflammatory disease**	1
***Triggered by medication***	2 (4.0)
Isotretinoin	1
Tocilizumab	1
***Infectious diseases***	3 (6.0)
Hepatitis C	2
HIV	1
***Other dermatosis except HS***	7 (34.0)
Acne conglobata	1
Erythema annulare centrifugum	1
Lupus erythematosus	1
Psoriasis	1
Subcorneal pustulosis	1
Sweet's syndrome	1
Vitiligo	1
**Pyoderma activity associated with comorbidity activity (n = 40)**	**6 (15.4)**
***Pathergy***	14
Grafting	1
Hepatectomy	1
Breast biopsy	1
Minor injury^a^	11
***Family history of associated diseases***	3 (6.0)
Crohn disease	2
Ulcerative colitis	1
***Treatment-related comorbidities***	
Arterial hypertension	11 (22.0)
Sepsis	7 (14.0)
Diabetes	5 (10.0)
**Substance use**	
Tobacco	14 (28)
Cocaine	2
Crack	1

a Dog Scratch, insect bite, hair removal, laceration, blunt trauma, piercing.

Pathergy was noted in 14 (28%) cases; in 3 cases after medical procedures (breast biopsy, hepatectomy and grafting), the remaining following minor injuries.

### Laboratory and histopathological tests

Laboratory data are shown in Table 4.

**Table 4:** Laboratorial and Histopathological aspects.

Aspects		n = 50 (%)	n_missing_
**Hemogram**			
Anemia		34 (68.0)	0
Hemoglobin value	Means (SD)	11.6 (2.2)	0
	Median (min‒max)	12 (6.3‒16)	
Leukocytosis		25 (50.0)	0
Leukocytes value	Means (SD)	12289.8 (6345.0)	
	Median (min‒max)	10815 (1108‒34200)	
**Inflammatory markers**			
High C-reactive protein		32 (78.0)	9
C-reactive protein value	Means (SD)	64.7 (86.6)	
	Median (min‒max)	22.8 (0.3‒354.3)	
Ferritin	Low	4 (10.8)	13
	Normal	19 (51.4)	
	High	14 (37.8)	
Ferritin value	Means (SD)	290.1 (387.7)	13
	Median (min‒max)	141 (7.4‒1944)	
Hypogammaglobulinemia		5 (13.5)	13
Gammaglobulinemia value	Means (SD)	1.30 (0.60)	13
	Median (min‒max)	1.20 (0.60‒3.60)	
**Histopathological**		n = 47 (%)	
Inflammatory infiltrate subtype	Lymphohistiocytic	4 (8.5)	3
	Neutrophilic	16 (34.0)	
	Mixed	27 (57.4)	
Granulomatous infiltrate associated		13 (27.7)	3
Local of the inflammatory infiltrate	Intraepidermal	14 (29.8)	3
	Dermal	46 (97.9)	3
	Hipodermal	18 (38.3)	3
	Anexial	7 (14.9)	3

SD, Standard Deviation; min, minimum value; max, maximum value.

Histopathological examination was available in 47 of the 50 cases, with a predominance of mixed inflammatory infiltrate (n = 27/47; 57.4%) followed by neutrophilic infiltrate (n = 16/47; 34%), granulomatous infiltrate (n = 13/47; 37.7%) and lymphohistiocytic infiltrate (n = 4/47; 8.5%). Detailed histopathological analysis is summarized in Table 4.

### Treatment

Prednisone was used in 44 out of 50 cases (88%), as the sole medication (n = 7/50; 14%) or in association (n = 37/50; 74%). The second most used drug was dapsone (n = 36/50; 72%). Of note, dapsone alone resulted in complete disease control in 2 cases. Fourteen patients (28%) used cyclosporine in addition to other medications and sixteen patients (32%) used at least one immunobiological agent (Table 5). Among these 16 patients using immunobiologicals, 5 achieved complete healing making it possible to gradually taper down and eventually suspend prednisone and other agents. In ten cases, disease control was achieved using immunobiologicals in association with other classical immunosuppressants (Table 6). Control was not achieved in one PG + HS patient despite the association of high doses of prednisone, cyclosporine, and two different immunobiologicals (adalimumab and ustekinumab).

**Table 5:** Medication (isolated).

Aspects	n = 50 (%)	n_missing_
Prednisone	44 (88.0)	
Cyclosporine	14 (28.0)	
Dapsone	36 (72.0)	
Doxycycline	5 (10.0)	
Colchicine	2 (4.0)	
Methotrexate	9 (18.0)	
Mycophenolate mofetil	5 (10.0)	
Azathioprine	13 (26.0)	
Immunobiologicals	16 (32.0)	
Infliximab	10 (62.5)	
Adalimumab	7 (43.8)	
Ustekinumab	4 (25.0)	
Secukinumab	2 (12.5)	
Belimumab	1 (6.3)	
Topical corticosteroid	14 (28.6)	1
Clobetasol	6	
Betamethasone	3	
Triamcinolone	2	
Clobetasol + triamcinolone	2	
Topical tacrolimus	9 (18.4)	1

**Table 6:** Drugs and associations.

Drugs and associations	Total healing	Disease control	No disease control	Total
Dapsone	0	2	1	3 (6.0)
Prednisone	5	2	0	7 (14.0)
Prednisone + dapsone	6	10	2	18 (36.0)
Prednisone + dapsone + cyclosporine	1	3	2	6 (12.0)
Immunobiologicals	2	1	0	3 (6.0)
Prednisone + immunobiologicals	1	2	0	3 (6.0)
Prednisone + dapsone + immunobiologicals	1	1	0	2 (4.0)
Prednisone + cyclosporine + immunobiologicals	0	0	1	1 (2.0)
Prednisone + dapsone + cyclosporine + immunobiologicals	1	6	0	7 (14.0)

Total	17 (34.0)	27 (54.0)	6 (12.0)	50 (100.0)

Of note, for 7 patients the main indication of immunobiological was PG; for 7 patients the main indication was IBD (5UC and 2CD); for one patient, psoriatic arthritis and for one patient, lupus arthritis (belimumab). Details about drugs, associations and outcome are described at Table 6.

### Hospitalization and progression

Hospitalization was required in 32 out of 50 cases (64%) in order to control either PG, pain or infection.

Disease control (healing of ulcers, enabling dose tapering of the main medication, usually prednisone) was achieved in 44 out of 50 (88%) patients after an average of 3.56 months (median 1‒34).

PG recurrence was observed in 49% of the cases, in most cases (70.8%) involving the same location of the initial lesion.

Complete disease remission occurred in 12 patients (24%), that are off therapy. Five patients required immunobiologicals to maintain disease control after total tapering of classic immunosuppressants. The median disease-free time was 37-months (12 to 192 months).

Patients using immunobiologicals were disease-free in 31.3% of cases, while patients not using immunobiologicals were disease-free in 35.3% of cases.

## Discussion

PG may occur at any age, especially between 20 and 50 years, and predominate in females.^1^ In this series, the mean age at diagnosis was 33.7 years and 58% of the patients were women, in accordance with the literature.^1^, ^2^, ^3^ Eight (16%) patients were younger than 18 at the time of disease onset.

In most cases (52%), PG was not the initial hypothesis, thus leading to a mean time from symptom onset to diagnosis of 26.5-months. These data demonstrate that PG still represents a diagnostic challenge possibly related to the rarity of the disease and multiplicity of differential diagnoses and being regarded as an exclusion diagnosis, despite its characteristic presentation.

Among the clinical subtypes, the ulcerative form is the most common^1^, ^3^, ^4^, ^5^, ^6^ and was also the most frequent in this analysis (86%). Lesions predominantly affected the lower limbs, corroborating with previous studies.^1^, ^4^, ^5^, ^8^, ^9^, ^10^

One of these cases had pulmonary involvement, which had been previously described in 42 case reports.^7^, ^8^, ^9^ Pathergy is described in 25% to 50% of the cases,^3^, ^5^, ^11^ and occurred in 24% of the studied patients.

The postoperative form of PG shows a lesser association with systemic diseases compared to other types of PG, with hematologic disorders being the most prevalent. The breast and abdomen are the most commonly affected areas in postoperative cases. On average, it develops around seven days after surgery. As it is often misdiagnosed as a wound infection, debridement can worsen the progression of the lesions. PG should be included in the differential diagnosis of postoperative wound dehiscence.^26^

Local pain, which is usually disproportionate to the size of the lesion, occurred in 78% of the present cases and 24% of the patients had a fever.^6^ Binus et al. observed pain in 62.1% of the cases in a retrospective study including 103 PG patients.^6^

Systemic diseases, mainly IBD, inflammatory arthropathies, and hematological disorders^6^, ^12^, ^13^ may precede, coexist, or follow the diagnosis of PG. Most of the patients (80%) had at least one associated condition. In this series, the most common non-dermatological condition was IBD (20%). PG may also occur in the context of a paraneoplastic syndrome. According to Shah et al., paraneoplastic PG usually presents as an ulcerative lesion on the extremities and is related to a breast tumor.^15^ Two of the patients had a history of solid neoplasia (liver and prostate).

One patient developed Sweet’s syndrome and 10 (20%) had HS. Six (12%) of the patients had associated autoinflammatory syndromes (PASH, PAPASH, PAPA and undefined autoinflammatory disease). The coexistence of PG and Sweet's syndrome has been reported.^19^, ^20^, ^28^, ^29^, ^30^, ^31^, ^32^, ^33^ Other dermatoses observed among the patients included: acne conglobata, lupus erythematosus (with lupus arthritis), psoriasis (with psoriatic arthritis), vitiligo, subcorneal pustulosis, and erythema annulare centrifugum.

In this series, 17 patients reported the use of tobacco (n = 14), cocaine (n = 1) and crack (n = 1). Keith et al. reported the association between PG and cocaine adulterated with levamisole. Cocaine has a toxic effect on endothelial cells and levamisole promotes vasculopathy, which are likely pathophysiological mechanisms in the development of PG.^21^, ^22^, ^23^ One patient developed PG after acne treatment with isotretinoin and 1 patient after anti-IL6 therapy (tocilizumab) for rheumatoid arthritis. Drug-induced cases are uncommon and may be related to an abnormal migration and functionality of neutrophils, triggering a dysregulated inflammatory response and apoptosis of keratinocytes.^19^, ^21^, ^22^

Anemia occurred in most of the patients (68%) due to several factors including associated diseases (IBD, hematological disorders), PG chronicity, bleeding from the lesions, dapsone-induced hemolysis. The authors also observed leukocytosis and increased CRP, which may be attributed to the PG pro-inflammatory status, the frequent association with secondary infection, and peripheral neutrophilia induced by systemic corticosteroids.^11^, ^23^

Intense neutrophilic infiltration was present in 34% of these cases, also affecting adnexa in 14.9%, and four of these cases (8.5%) had lymphohistiocytic infiltrate. Granulomatous infiltrate may also occur^1^ and it was observed in 27.7% of these cases. Few studies have evaluated the histopathological features of PG. Chakiri et al. reported that among 14 patients with PG, a dense neutrophilic infiltrate occurred in all cases, with vasculitis in 4 cases and lymphoplasmacytic infiltrate in 5 cases.^1^ The histopathologic findings of PG are not specific and vary according to the stage of the lesion. Initial lesions may show deep suppuration, often folliculocentric, with dense neutrophilic infiltrates.^1^, ^11^ After ulceration, there may be necrosis and hemorrhage, thrombosis of dermal or hypodermic blood vessels, with a lymphocytic infiltrate.^11^

PG treatment aims to reduce inflammatory activity and pain, promote wound healing, and control associated diseases.^2^, ^11^, ^24^, ^25^ It still poses a challenge as no specific therapy is currently available and there is no consensus on which treatment is most effective. Systemic corticosteroids are generally effective and considered the first-line treatment at a dose equivalent to 0.5–1 mg/kg/day of prednisone. More resistant lesions require longer therapy (>3-months), increased doses, or association with other immunomodulatory agents. In the present study, most cases were treated with prednisone 0.25‒1.5 mg/kg/day (88%) as primary line therapy.^1^, ^2^, ^4^, ^5^, ^6^, ^11^, ^24^ Cyclosporine is also considered a first-line therapy often in association with prednisone in recalcitrant cases and was required in 28% of these cases at some point in the follow-up.^27^

Other immunosuppressants and immunomodulators may be indicated, such as dapsone, azathioprine, mycophenolate mofetil, cyclophosphamide, methotrexate. Immunobiological agents including infliximab, adalimumab, ustekinumab and canakinumab have also been utilized.^1^, ^2^, ^4^, ^5^, ^6^, ^11^, ^24^ The second most used drug in our series was dapsone (72%) and two out of three patients achieved disease control with dapsone in monotherapy. Azathioprine was also used in 13 of our patients for the treatment of IBD, however, it showed no improvement in PG lesions.^1^, ^2^, ^4^, ^5^, ^6^, ^11^, ^24^

Immunobiologicals are promising therapeutic options due to their activity on inflammatory cytokines involved in the pathogenesis of PG, such as TNF-α, IL-1, IL-17.^2^, ^4^, ^24^, ^27^^,^^28^ Sixteen of our patients (32%) used at least 1 immunobiological agent once PG lesions were refractory to corticosteroid therapy (n = 9) or for the management of a comorbidity (n = 7). It has been demonstrated that some IBD patients have an adequate response only with immunobiologicals, suggesting that they should be considered as first-line therapy for the treatment of PG.^8^, ^11^, ^12^

Most patients required hospitalization at least once (32 cases, 64%), similar to the rate reported by Platzer et al. in a review of 36 cases (69.4%).^4^ Seven (14%) of our patients developed sepsis during immunosuppressive therapy in at least one of the disease recurrences. Among these seven patients, 3 used prednisone + dapsone + cyclosporine + immunobiologicals; 1 used prednisone + dapsone + immunobiologicals; 1 used prednisone + immunobiologicals; and 2 used only prednisone. Infections are among the main causes of death in PG studies.^4^, ^13^ Two of our patients presented severe cataracts as a complication of prolonged use of prednisone. One patient developed irreversible nephropathy and demyelinating disease after prednisone and cyclosporine. These data may suggest the benefits of early introduction of immunobiologicals.

Disease control was achieved in 44 out of 50 patients (88%) after an average of 3.56 months, in agreement with the remission rates described.^3^ PG recurrence occurred in 49% of our patients, mostly in the previous location (70.8%). Variable recurrence rates were reported in the literature (17%‒61%) and were also more common in the primary lesion site.^3^, ^4^

At the end of our follow-up, among 17 patients (34%) that were disease-free, five patients remained using immunobiologicals while 12 patients were off any maintenance therapy. All 5 patients who achieved complete disease remission with immunobiological had IBD (3UC and 2CD) and 3 also had HS. None of the 5 had a recurrence of lesions. These data provide additional evidence that patients with comorbidities may benefit from the early use of immunobiologicals.^8^, ^11^, ^12^ Of note, 20 of 32 (62.5%) patients with idiopathic PG or HS-associated or syndromic PG controlled with classic immunosuppressants, and 6 (18.75%) of them only achieved disease control with immunobiologicals.

The main limitations of the present study are its retrospective nature, the descriptive design, and the number of patients. Therefore, although it seems like a small sample compared to more common diseases, PG is a rare disease, and this is one of the largest series ever published including Latin America and patients and with a long follow-up.

## Conclusion

The present study confirms that PG is a rare disease, often difficult to diagnose and associated with systemic diseases, and autoinflammatory syndromes. Although systemic corticosteroids remain a first-line therapy, this data suggests that expanding the use of immunobiologicals early in the management of PG may represent a promising therapeutic tool to avoid disease recurrences and frequent hospitalizations.

## Financial support

None declared.

## Authors’ contributions

Livia Maria Oliveira Salviano: Approval of the final version of the manuscript; critical literature review; data collection, analysis and interpretation; effective participation in research orientation; intellectual participation in propaedeutic and/or therapeutic management of studied cases; manuscript critical review; preparation and writing of the manuscript; statistical analysis; study conception and planning.

Denise Miyamoto: Approval of the final version of the manuscript; effective participation in research orientation; intellectual participation in propaedeutic and/or therapeutic management of studied cases; manuscript critical review; study conception and planning.

Claudia Giuli Santi: Approval of the final version of the manuscript; effective participation in research orientation; intellectual participation in propaedeutic and/or therapeutic management of studied cases; manuscript critical review; study conception and planning.

Tatiana Mina Yendo: Approval of the final version of the manuscript; effective participation in research orientation; intellectual participation in propaedeutic and/or therapeutic management of studied cases; manuscript critical review; study conception and planning.

Maria Cecilia Rivitti-Machado: Approval of the final version of the manuscript; effective participation in research orientation; intellectual participation in propaedeutic and/or therapeutic management of studied cases; manuscript critical review; study conception and planning.

## Conflicts of interest

None declared.
